# Vitreous Diagnosis in Neoplastic Diseases

**DOI:** 10.1155/2012/930704

**Published:** 2012-09-26

**Authors:** Mónica Asencio-Duran, José Luis Vallejo-Garcia, Natalia Pastora-Salvador, Agustín Fonseca-Sandomingo, Mario R. Romano

**Affiliations:** ^1^Hospital La Paz, Madrid, Spain; ^2^Istituto Clinico Humanitas, Milano, Italy

## Abstract

Vitreous body is an intraocular structure, origin of diverse pathologies, but is also the place where cells and inflammatory mediators are released coming from several pathologic processes. These inflammatory reactions can happen in any other ocular location like choroid, retina, optic nerve, or ciliary body and vitreous humor constitutes a stagnant reservoir for these resulting substances and debris. Through the recent techniques of vitreous collecting, handling, and analysis, increasingly more sophisticated and with fewer complications, cellularity and molecules in the vitreous of challenging pathologies for the ophthalmologist can now be studied. The most usefulness for vitreous diagnosis would be the masquerade syndromes, and the best exponent in this group is the primary vitreoretinal lymphoma (PVRL), in which cytology and an IL-10/IL-6 ratio more than 1 is fundamental for the diagnosis.

## 1. Introduction

Vitreous body is the clear gel that fills the vitreous chamber or posterior chamber (PC) of the eyeball, the space between the lens and the eyewall, whose inner layer is the neurosensorial tissue that receives and transmits the image to the central nervous system called the retina. Its functions are to give volume to the eye, to support the retina attached, and to maintain its transparency to allow light beams to reach onto the retina. 

Unlike the fluid in the anterior segment of the eye (aqueous humour), which is continuously replaced, vitreous humor is stagnant, and its composition remains quite constant throughout life. The vitreous gel is avascular, composed mainly of water (98-99%), and 0.9% of inorganic salts (sodium, potassium, and chloride). The remaining 0.1% is divided between protein, polysaccharide components, and ascorbic acid. Most of the protein is forming fibrils composed of a small collagen type V/XI core wrapped in a thick layer of collagen type II (75% of the fibril by mass) [1, 2]. It also contains very few cells, mostly phagocytes, whose function is to remove undesired cellular debris from the visual field, as well as hyalocytes of the surface of vitreous, which act as macrophages [3, 4].

The vitreous is feebly antigenic and is characterized by the absence of gamma-globulins and immunocompetent cells [5]. Because it only exhibits phagocytosis, this represents an incomplete and primitive immunological system, reacting like an embryonic tissue. The immune privilege, also, a physiologic mechanism characteristic of the internal compartments of the eye, is designed to provide protection against pathogens, protecting the delicate visual axis from the sight-destroying effect of immunogenic inflammation [6]. At the same time, there is a sustained suppressive microenvironment in the PC of the eye that inhibits the local expression of preexisting systemic immunity and participates in modifying the primary immune responses to ocular antigen [7, 8]. In the vitreous cavity it would develop a deviant form of immunity, similar to that in the anterior chamber [9], in which antigen-specific suppressor T cells are generated and delayed hypersensitivity reactivity is selectively impaired. Both the vitreous and the retina supply immunosuppressive molecules to the PC, but it has been suggested that retinal cells contribute more significantly to the immune suppressive microenvironment than vitreous cells: TGF *β* is produced by retinal astrocytes and retinal pigment epithelium; and Muller's cells (glia) of the retina suppress T cell proliferation by a direct contact mechanism. In addition, the retinal vascular endothelium, Bruch's membrane, and the pigment epithelium together form the so-called ocular-blood barrier [7].

## 2. Ocular Diseases with Clinical Repercussion in Vitreous Body

Vitreous has a major role in the origin and the triggering of several ocular pathologies. Posterior vitreous liquefaction developed through years by means of dissolution of collagen fibers yields to several primary degenerative pathologies in vitreoretinal junction. Other diseases are easily diagnosable in the fundus eye and only affect the vitreous in late stages, like retinal vasculopathies as diabetic retinopathy and other vitreoretinal proliferations. Also, certain eye diseases have their beginning in other more hidden structures of the eye but may secrete molecules or even cells to the vitreous chamber, causing symptoms and helping in the diagnosis, since the vitreous is more accessible to study than other posterior pole structures. 

The latter is the case of uveitis, a wide term that actually comprises a large group of diverse diseases affecting the retina, optic nerve, and also the vitreous compartment. These diseases may affect, in addition, several territories of the eye simultaneously and can have in major or minor degree manifestation in the vitreous humor, specially those concerning the posterior chamber. Fifty percent of noninfectious or “autoimmune” cases are limited to the eye (organ specific), whereas the remainder forms part of more generalized diseases, so that the pathophysiology of uveitis depends on the specific etiology, but in all types there is a breach in the ocular-blood barrier that normally prevents cells and large proteins from entering the eye. These cells then recognize antigens (autoantigen or foreign antigen) presented on the cell surface of antigen-presenting cells (APCs, like dendritic cells or macrophages), and activation and clonal expansion will take place, which results in increased production of IL-2, interferon-*γ*, and TNF-*α* [10]. Only certain responses are capable of overcoming the condition of immune privilege of the eye and unleashing the inflammatory cell accumulation and the tisular damage. 

In most cases, the clinical appearance is sufficient for diagnosis, but since the majority of these patients have an unknown etiology for the intraocular inflammation, and this can be in addition related to a primarily nondiagnosed systemic disease, could be infectious, inflammatory, or even tumoral, the correct diagnosis can prove difficult. Thus, the intraocular inflammation is associated with the increased expression and action of several cytokines and growth factors, which can be determined in the vitreous and can help in the diagnosis. Several molecules have been identified along the last decades in the vitreous humor, which may be the key in the physiopathology of certain ocular diseases. In most cases this determination has investigational purposes, in others is helpful for the prognosis of the patients, and in a minority of cases its finding constitutes an indispensable diagnostic tool.

## 3. Diagnostic Techniques in Atypical ****Presentations of Uveitis

Many patients with uveitis have such characteristic ocular signs and symptoms, associated systemic disorders, and laboratory abnormalities that a satisfactory clinical diagnosis can be established without the need for invasive intraocular studies. Most other patients have mild, self-limited, and/or readily controllable disease that does not warrant aggressive invasive testing. In contrast, some patients have atypical ophthalmic and/or systemic features or do not respond to conventional anti-inflammatory therapies. Several techniques have been employed for diagnostic purposes in these cases: aqueous aspiration, vitreous aspiration, diagnostic vitrectomy, fine-needle aspiration biopsy, controlled aspiration of subretinal fluid, incisional chorio-retinal biopsy, and diagnostic enucleation.

### 3.1. Aqueous Aspiration

The indications of anterior chamber aspiration may be varied, but most common situations are patients with anterior chamber inflammation with suspicion of masquerade syndromes, a hypopion suspicious of infection, endophthalmitis, lens-induced uveitis and for cytopathologic examination [11]. A fine needle (30, 27, or 25 gauge) is introduced with the bevel up through clear cornea over the iris stroma, with optimal visualization by means of slit lamp or the surgical microscope, taking care to avoid the lens. A 0.1-0.2 mL of aqueous humor is withdrawn into a 3 mL syringe in a sterile technique, and then balanced salt solution may be used to reform the chamber [12]. The cytospin technique or others can be used to increase the sensitivity of the cytology specimen [13]. In the aqueous humor many other techniques can be performed for the diagnosis of infectious posterior uveitis, as polymerase chain reaction (PCR) and pathogen-specific antibody production for herpes simplex virus (HSV), varicella zoster virus (VZV), cytomegalovirus, Epstein-Barr virus (EBV), human immunodeficiency virus (HIV), Propionibacterium acnes, and Toxoplasma gondii [14]. Further studies have demonstrated for PCR of aqueous humor to yield a diagnosis in one-third of patients with posterior infectious uveitis with a sensitivity of 82% and specificity of 100%, equal or better than vitreous biopsy [15]. The advantage of anterior chamber aspiration is that it can be performed in an outpatient setting, but the disadvantage is that it retrieves a limited sample volume of 100 to 200 *μ*L per procedure [16], which limits the number of molecular examinations that can be performed on the sample.

### 3.2. Vitreous Aspiration Tap

The indications for this technique are basically in posterior uveitis unresponsiveness to treatment when malignancy needs to be ruled out; when intraocular infection is considered the primary cause of inflammation in the absence or insignificant amount of vitreous cells that would preclude diagnosis through pars plana vitrectomy (PPV) [17, 18]. The major indication for vitreous aspiration would be when intraocular lymphoma is a significant diagnostic possibility. The technique of vitreous aspiration is similar to that of anterior chamber paracentesis and is currently performed through the pars plana under local anesthesia with a large caliber needle, such as 21-gauge (G) hollow needle or fine, like 23 or 25-G mounted on a 1-mL syringe as an aspirating device, permitting to aspirate a volume of 100 to 250 *μ*L of vitreous humor while the needle is directed posteriorly towards the optic nerve head. With vitreous biopsy, specimens obtained using a needle and syringe were positive in 54% of infected eyes compared with 75% of the specimens collected with vitrectomy procedures [18], although posteriorly, in the Endophthalmitis Vitrectomy Study, no significant difference was shown between needle tap versus mechanized vitreous biopsy with respect to microbiologic yield [19]. The main complications associated with the technique are retinal tears and endophthalmitis; however the risk is low, but both more common than after vitrectomy [20]. Other advantages of vitreous biopsies over vitrectomy are quickness, can be done in the outpatient setting without inpatient admission, can be repeated, and are less traumatic to the eye [20]. 

**Figure 1:**
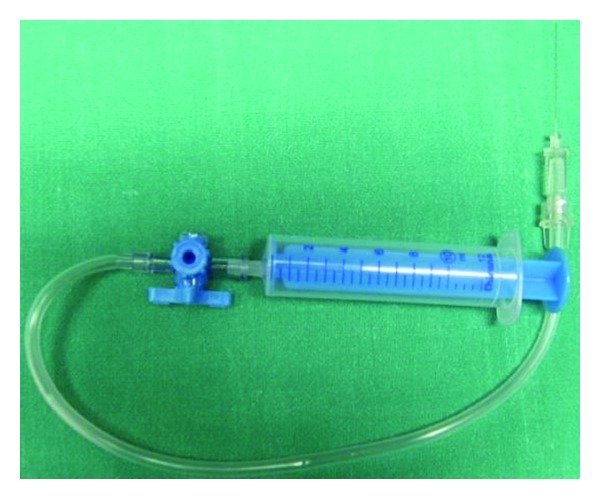
Set for manual Fine-needle aspiration biopsy/cytology, composed of 10 mL syringe, extension tube with a three-way stopcock, and long 27-gauge needle.

Another variation of the technique is the fine-needle aspiration biopsy (FNAB), in which a fine-needle gauge is directed to the localized suspected areas of intraocular tumor or lesion. Aspiration is performed automatic or manually using a 25-G to 30-G needle connected to an aspirating syringe (Figure 1). In case of intraocular tumor, the aspirated block obtained is likely to have a higher concentration of neoplastic cells than any of the adjacent intraocular fluids, decreasing the possibility of inconclusive cytological diagnoses, which often occur in vitrectomy specimens. For aqueous or vitreous humor the technique turns into an effective method for cytology that generally is able to obtain a sufficient amount of cells (100 to 500 *μ*L of ocular fluid) to perform routine analysis like microbiologic, cytomorphological evaluation of Papanicolaou or haematoxylin- and eosin-stained cells, immunocytochemical analysis, and other applications [21]. The fine-needle aspiration technique is less invasive and has fewer complications than others [22], being in certain centers the preferred method for diagnostic purposes.

### 3.3. Diagnostic Vitrectomy

This technique may be the better option in selected cases, such as when vitreous removal is considered to be not only diagnostic but also therapeutic (e.g., endophthalmitis, intraocular bleeding with suspected malignant origin, and for the treatment of complications of chronic uveitis), and when the eye is inflamed and thereby patients may experience substantial discomfort during the vitreous biopsy tap [23]. Some authors recommend that vitrectomy-assisted biopsy should be considered only in cases in which FNAB fails [22], or multiple tests are needed and therefore requiring several punctures. A diagnostic standard three-port pars plana vitrectomy (VPP) provides a large amount of vitreous, retina, or choroid (though diluted), but always requires an operation theater under sterile conditions and direct visualization of the vitrectomy instruments. In order to obtain an undiluted vitreous sample, the infusion cannula of the system must be closed and the vitreous specimen is collected through undiluted lines using the vitreous cutter connected directly to a 3 mL syringe until the eye is noted to soften visibly [24]. At least 1.5 mL of undiluted vitreous can be reliably obtained with this technique. With perfluorocarbon-perfused vitrectomy, in which aspirated vitreous is compensated with perfluorocarbon liquid entry during vitreous aspiration, other authors were able to obtain an average of 2.4 mL of undiluted vitreous [25].

There is controversy whether using classic 20-G ports VPP needing suture, or the newest microincisional systems with 23-, 25-, or 27-G systems, but with anyone of these, the overall diagnostic yield of VPP varies considerably in different published studies from 14.3 to 61.5% [26–31], and the success for the procedure was greater when an intraocular infection was suspected compared with an intraocular malignancy [31], and greater for detecting primary vitreoretinal lymphoma than for detecting metastatic disease.

### 3.4. Chorioretinal Biopsies

Biopsies have been performed to investigate uncertain uveitis, choroiditis, and retinal and choroidal masses [32]. The Indications for biopsy included major diagnostic uncertainty, suspected cancer metastasis to the choroid without other evidence of systemic malignancy, and patient insistence on biopsy confirmation of the diagnosis prior to treatment. The procedure may be performed transsclerally or by an ab interno approach. Fine-needle aspiration biopsy is another method of obtaining retinal and choroidal tissue [33]. The limited performance of intraocular biopsy is explained by the risks for dissemination of malignant cells, eye complications (mainly hemorrhage, retinal detachment, and infection), and fears of misdiagnosis, although the literature gives little support to these [34, 35]. However, on the other hand, several authors have claimed that identifying patients with aggressive disease and a high risk for dissemination in malignant processes should be a priority and histopathological diagnosis should be mandatory [36, 37].

Various techniques have been developed to minimize the risks aforementioned. In the classic, transscleral approach, a scleral flap is created. A sharp blade then incises the choroid, and the biopsy tissue is grasped with forceps. A retinal specimen may also be obtained with the choroidal specimen if a chorioretinal sample is the subject of study. Several modifications have been later described to facilitate the biopsy procedure and also reduce the risk of complications [38]; VPP is now often performed before creating the scleral flap, and another modification is the use of cyanoacrylate glue to provide increased stability to the tissue. In the transvitreal or ab interno approach, a retinochoroidectomy down to the sclera is performed after vitrectomy. The risk for complications is high, mostly due to hemorrhage and retinal detachment. FNAB for choroidal lesions provides the least invasive method of harvesting tissue [39]. Anterior lesions (iris and/or ciliary body) may be approached via limbal entry. The pars plana approach provides access for posterior lesions. The tip of the needle may be bent, facilitating entry into shallow choroidal lesions. Also, the risk of posterior scleral perforation is decreased.

## 4. Vitreal Biomarkers of Uveitis

Studies have shown increased levels of IL-6 (T-cell cytokine) in the vitreous fluid of patients with active intermediate or posterior uveitis, although it did not correlate with a specific uveitis type [40], suggesting that IL-6 is an inflammatory mediator common in various uveitis etiologies. IL-12, produced by monocytes, macrophages, B cells, and connective tissue-type mast cells has, also been found increased in aqueous humor and vitreous fluid of patients with low-grade intraocular inflammation and in uveitis in clinical remission for as long as 2 years [41]. Intraocular inflammation that fails to respond to immunosuppressive treatment raises suspicion for another different process. Since diagnostic analysis of vitreous fluid in patients with uveitis is limited, the best challenge for the study would be the masquerade syndromes.

## 5. Uveitis Masquerade Syndromes

**Figure 2:**
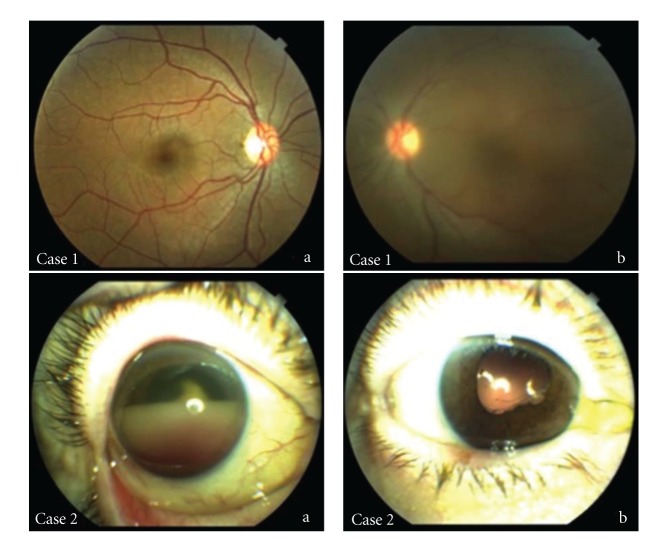
Case 1: (a) healthy right eye of the same patient, (b) left eye vitreitis in a healthy patient that was finally diagnosed of primary vitreoretinal lymphoma by vitrectomy. Case 2: (a) hemorrhagic hypopion in a patient with primary thoracic B-cell Hodgkin's lymphoma in clinical remission. After unsuccessful anti-inflammatory and antibiotic treatment an anterior chamber paracentesis was performed confirming the diagnosis of metastatic lymphoma, (b) external aspect of the eye after treatment with intravitreal methotrexate.

*Uveitis masquerade syndrome* (UMS) is a group of disorders that mimic intraocular inflammation, but cells seen may be of noninflammatory origin (e.g., pigment, blood or malignant cells) or are inflammatory but secondary to another disorder [42, 43]. Theodore in 1967 was the first author who described a conjunctival carcinoma manifesting as a chronic conjunctivitis and named it *masquerade syndrome* [44]. The frequency of UMS among the patients with uveitis in a tertiary ophthalmologic center was 5% [45]. The causes of UMS may be variate, such as malignant, including hematologic malignancies, retinoblastoma, melanoma, and lung cancer metastasis; or nonmalignant, like ocular toxoplasmosis, diabetic retinopathy, hypertension, retinal detachment or degeneration, intraocular trauma, and radiation retinopathy [42, 43, 45–69] (Table 1). They are often misdiagnosed as a chronic idiopathic uveitis, but they can present in any location of the eye manifesting as panuveitis, pars planitis, vitreitis, papillitis, anterior segment cells, hypopyon or vitreal, and/or chorioretinal infiltrates (Figure 2).

Although they constitute rare presentations of uncommon diseases in the eye, the ophthalmologist must be aware because many of the UMS etiologies are malignancies with deleterious effects for the patient, for what early diagnosis and prompt treatment are mandatory. The study of vitreous body can be of great help specially in these cases, since the achievement of a small sample in doubtful cases can provide us the diagnosis.

### 5.1. Intraocular Lymphoma

#### 5.1.1. Classification

Although both Hodgkin's lymphoma and non-Hodgkin's lymphoma (NHL) can present as intraocular inflammation, in the case of Hodgkin's lymphoma ocular involvement generally is rare and often occurs late in the course of the disease, whereas NHL affects more commonly the eye. NHL can be divided in two clinically different entities: systemic NHL with metastases to the eye, and NHL of the central nervous system (NHL-CNS). Recently, Coupland and co-workers proposed an anatomical classification according to the localization of the disease in the eye; retinal lymphomas are high-grade B-cell malignancies associated with a poor prognosis, whereas primary uveal lymphomas are typically low-grade B-cell tumours derived from the postgerminal centre (memory) B cell [70].

The variant with major ophthalmic repercussion is the primary vitreoretinal lymphoma (PVRL), a subtype of primary central (CNS) lymphoma, typically classified as a diffuse large B-cell lymphoma and most frequently develops in elderly populations. Over 15% of primary CNS lymphoma patients develop intraocular lymphoma, usually occurring in the retina and/or vitreous, and conversely, 65%–90% of PVRL patients develop CNS lymphoma [71]. Consequently, PVRL is often fatal because of ultimate CNS association, that can appear from 1 month to 10 years after [72, 73]. 

#### 5.1.2. Clinical Features

Both retinal and uveal lymphoma can manifest as any form of uveitis, but PVRL typical clinical findings include vitreous cellular infiltration (lymphoma and inflammatory cells) and subretinal tumor infiltration. Choroid is the predominant location for primary uveal lymphomas and most often manifests as recurrent episodes of blurred vision and metamorphopsia secondary to exudative retinal detachment affecting the fovea. A classic finding is the presence of solitary or multiple yellow, creamy choroidal infiltrates with clear vitreous, that can evolve to diffuse thickening of the uveal tract and in some cases, to episcleral extension appearing as a nonmobile orange to yellow or “salmon” patch.

#### 5.1.3. Sample Collection and Handling

The clinical suspicion is very important given the potential lethality if an uncorrect diagnosis is made and a proper systemic treatment is applied. Currently, PVRL is most often diagnosed using citology (the gold standard) or vitrectomy to identify lymphoma cells in the vitreous or retina [74]. In order to prevent degeneration of lymphoma cells, vitreous specimens are placed into a tube containing culture medium like RPMI (Roswell Park Memorial Institute) [75], whereas others prefer immediate placement in normal saline, taking care not fixing with alcohol with the aim to not alter the identification of PVRL cells in the vitreous sample. 

#### 5.1.4. Sample Analysis

**Citology**

As lymphoma cells are fragile, the general consensus recommends sending the samples immediately to an experienced cytopathologist to distinguish the malignant cells (usually B lymphocytes) from the reactive lymphocytes (T cells). The malignant B cells of PVRL exhibit characteristic features with Papanicolaou, Giemsa, or Diff-Quick stains [26, 76]: large round or oval nuclei, frequently segmented and often containing prominent nucleoli, surrounded by scant basophilic cytoplasm. Samples often are negative because of poor biopsy samples, with a reported effectivity of only 48.3% of lymphoma cases for PPV, although other authors have found for FNAB a diagnostic effectivity in 87.5% of the suspicious intraocular lymphoma cases [17]. Other authors advocate for the fixation of the samples with Cytolit or HOPE solution (Herpes-glutamic acid buffer mediated organic solvent protection effect) in order to facilitate the transportation from the theater to the laboratory [77].

**Molecular Analysis**

Flow cytometric immunophenotyping (FCI) can be done in diluted samples, allows for the analysis of several different cell surface markers simultaneously, and offers a quantitative method of determining the percentage of a particular cellular phenotype, increasing the efficiency of a biopsy specimen [26]. Dilute vitreous is centrifuged and resuspended in cell culture medium, and cells are counted and stained with antibodies to detect markers that identify leukocytes, T lymphocytes, B lymphocytes (including CD19, CD20, CD22, *κ*, and *λ* light chain markers), monocyte/macrophages, and lymphocyte activation. The test relies on the finding that the majority of PVRL have restricted expression of *κ* or *λ* chains, with the most sensitive marker being a *κ* : *λ* ratio ≥3 or ≤0.6 (80%), whereas CD22 and CD20 markers are not very sensitive for lymphoma (50 and 33%, resp.), although they are quite specific (94 and 89%, resp.) [31]. For patients with possible T-cell lymphoma, cell surface markers more commonly searched are CD3, CD8, CD4, CD7, CD2, CD25, and CD52 [78].

Other molecular analysis techniques like microdissection and PCR can be used. Microdissection allows for the selection of only few or poorly preserved malignant or atypical lymphoid cells that would have been nondiagnostic for PVRL by routine cytological techniques. PCR can determine monoclonality by immunoglobulin heavy chain (IGH) rearrangement and t(14; 18) translocation of the bcl-2 gene that promote cell survival and predict a more aggressive tumor course in B-cell lymphoma [75, 79, 80]. PCR has been found to be 64% sensitive for PVRL [81], and is being used to study the genotypic classification of PVRL with the goal of identifying prognostic factors; patients with a translocation in the bcl-2 gene are significantly younger than patients who lacked the translocation, suggesting that younger patients with the translocation may need to be treated aggressively [82]. Some authors have advocated an inhibition of B-lymphocyte chemoattractants (BCA-1, CXCL13, and SCYB13) and their ligands CXCR4 and CXCR5 could be a future strategy for the treatment of this disease with limited side-effects profile [83].

For Margolis, vitrectomy together with cytology and flow cytometry detected all cases of PVRL [84]. If the quality of the cytology finally is poor, then a second vitrectomy may be necessary, but because cell numbers are likely to be low in a vitrectomized eye, a retinochoroidal biopsy may be performed at the time of vitrectomy surgery [85].

**Vitreous Biomarkers of Intraocular Lymphoma**

Possibly, PVRL is the best example of ocular disease in which intravitreal cytokines are more useful for the diagnosis. Increased concentration of IL-10, a growth and differentiation factor for activated B lymphocytes, has been found increased in vitreous fluids of PVRL patients [75], in contrast with the increased concentration of IL-6 characteristic of uveitis, for which many authors have indicated that an IL-10/IL-6 ratio greater than 1.0 is useful for the diagnosis of PVRL. Cytokine analysis can be useful adjunctive tests in corroborating suspicion of PVRL and determining whether there is a significant response to treatment [75, 80–87], but cannot be used only to make the diagnosis, as some studies have reported false positive or false negative results [88]. The IL-10/IL-6 ratio greater than 1.0 in suspected cases of PVRL was associated with a sensitivity and specificity of 74.3 and 75.0%, respectively, [86] and Cassoux and co-workers found in 51 vitrectomies performed in patients with proven PVRL that an IL-10 cut-off value of 400 pg/mL was associated with 80% sensitivity and 99% specificity [89].

The diagnosis of intraocular lymphoma from vitreous specimens depends on proper handling of the specimens, methods of aspiration, concentration, fixation, and staining [90, 91]. Addition of culture medium with fetal calf serum can improve the survival and viability of the malignant cell [91]. Prior treatment of patients with steroids reduces the number of viable lymphoma cells, which are known to be cytolytic, so that discontinuing systemic and topical corticosteroids is strongly recommended before biopsy to increase the profitability of these cells [91].

### 5.2. Other Lymphoproliferative Malignancies

Leukemia has increased the variability of ocular presentations associated, due to the improvement in the survival after the new era of effective antileukemic therapy. Leukemia may involve almost every ocular tissue, with the retina being the most frequent affected structure (up to 69% of all patients show fundus changes at some point in the course disease). Hemorrhages, infiltrates, and aggregates of leukemic cells are found at all levels [48, 92], and generally the internal limiting membrane acts as a barrier; however, cells occasionally invade the vitreous possibly emerging from the optic nerve head and these cases can be diagnosed by examination of the specimens obtained from the vitreous [93, 94]. Nevertheless, primary presentation of these diseases are rarely ophthalmological and more frequently occur in patients with advanced systemic disease. As relapsing uveitis or hyphemas can be related to leukemia, are in these cases when cytological sample must be obtained, thus allowing us to ascertain the inflammatory origin or reactivation of the disease.

### 5.3. Uveal Melanoma

Uveal melanoma (UM) is the most frequent intraocular tumor in the adulthood. Funduscopy combined with ultrasonography actually gives an accurate diagnosis in almost 95% of the patients, but there are, however, some cases difficult to diagnose due to atypical ocular manifestations or accompanying intraocular changes, such as extensive retinal detachment, vitreous hemorrhage, or others. In these cases histopathological examination with preservation of the eyeball is the ideal method. 

Cytological tests using modified Shorr's or others stains have been capable of diagnosing cells with intracytoplasmic melanin pigment granules from samples obtained in eyes harbouring choroidal or metastatic cutaneous melanomas [95, 96]. A recent study shows that 5-S-cysteinyldopa (5-S-CD), a metabolite generated during pheomelanin synthesis, may reflect a direct secretion from the tumor into the vitreous or an alteration of dynamics of intraocular fluids, because its concentration is increased in vitreous fluid from UM patients. But the diagnosis from vitreous samples in UM probably would not become extensible in the future due to the unknown exact role of this biomarker [97], the possibility of extraocular dissemination of UM implicit in the surgical intervention [98], and to the efficiency of other simpler diagnostic methods.

### 5.4. Intraocular Metastasis

Intraocular metastases often appear in the choroid as solitary or multiple mass in a patient with history of systemic malignancy, although in 34% of the cases had no known primary site [99]. Together with the possibility of bilateral involvement and atypical clinical presentations, the diagnosis sometimes is difficult. Vitrectomy has helped to diagnose metastatic cutaneous melanoma in difficult cases like nonpigmented vitreous clumps [100] and thickened posterior vitreous membranes [101], and moreover can be therapeutic in these cases. Carcinomas also can metastasize directly in the vitreous or indirectly by means of vitreal seeds from an underlying choroidal, retinal, or optic nerve infiltration, and both vitrectomy or fine-needle aspiration cytology can help in the diagnosis [102, 103].

## 6. Conclusion

Vitreous body constitutes a little-known intraocular structure, but we are increasingly supporting our diagnostic searches in it thanks to the recent advantages in collection, handling, and analysis of vitreous samples. Inflammation is not always the cause of apparent inflammatory diseases and sometimes the origin is degenerative, traumatic, vascular, infectious, or even neoplastic. Vitreous cells in hands of experienced cytologists can be sufficient, but an accurate diagnosis needs the employment of sophisticated molecular analysis such as flow cytometric immunophenotyping (FCI), microdissection and polymerase chain reaction (PCR), or cytokine analysis like IL-10/IL-6 ratio. The major diagnostic use of vitreous sampling would be the masquerade syndromes, in which a devastating neoplastic disease can be behind a few vague and slightly specific ocular signs, and possibly the biggest representative of this group is the primary vitreoretinal lymphoma and some metastatic intraocular lesions. Even though, little information exists nowadays on the number and the specific role of different molecules acting in the pathophysiology of diseases that represent a challenge for our daily practice. Further investigation is needed to increase our knowledge on the molecular pathogenic mechanisms underliying neoplastic diseases, with which we could interact to create new targeted and powerful therapeutic pathways or at least alternatives to the current ones. 

## Figures and Tables

**Table 1:** Ophthalmic diseases masquerading as chronic idiopathic uveitis.

Malignant diseases	
Intraocular lymphoma	
Non-Hodgkin's lymphoma of the central nervous system	
(NHL-CNS)	
Systemic Non-Hodgkin's lymphoma metastatic to eye	
Hodgkin's lymphoma	
Other lymphomas	
Lymphoid hyperplasia of uvea	
Leukemia	
Carcinoma metastatic to the eye	
Uveal melanoma	
Childhood malignancies	
Retinoblastoma	
Coats' disease	
Leukemia	
Medulloepithelioma	
Juvenile xanthogranuloma	
Paraneoplastic syndromes	
Cancer-associated retinopathy	
Melanoma-associated retinopathy	
Bilateral diffuse uveal melanocytic proliferation	

Nonmalignant diseases	

Multiple sclerosis	
Intraocular foreign body	
Vascular disorders (hypertension, diabetic retinopathy, radiation retinopathy, retinal vasculitis, branch/central vein occlusion, ocular ischemic syndrome)	
Retinal detachment	
Vitreous and retinal degenerations (myopic, tapetoretinal)	
Pigment dispersion syndrome	
Intraocular infections (bacterial, fungal, viral, parasitic, propionibacterium acnes)	
Postvaccination and drug-related reactions	
